# Predicting intention to treat HIV-infected patients among Tanzanian and Sudanese medical and dental students using the theory of planned behaviour - a cross sectional study

**DOI:** 10.1186/1472-6963-9-213

**Published:** 2009-11-20

**Authors:** Anne N Åstrøm, Elwalid F Nasir

**Affiliations:** 1 Institute of Clinical Dentistry, Faculty of Medicine and Odontology, University of Bergen, Bergen, Norway; 2 Faculty of Dentistry, University of Science and Technology, Khartoum, Sudan

## Abstract

**Background:**

The HIV epidemic poses significant challenges to the low income countries in sub Saharan Africa (SSA), affecting the attrition rate among health care workers, their level of motivation, and absenteeism from work. Little is known about how to deal with deterioration of human resources in the health care systems. This study aimed to predict the intention to provide surgical treatment to HIV infected patients among medical- and dental students in Tanzania and Sudan using an extended version of the Theory of Planned Behaviour (TPB).

**Methods:**

Four hundred and seventy five medical- and dental students at the University of Dar es Salaam (mean age, 25 yr) and 642 dental students attending 6 public and private dental faculties in Khartoum (mean age 21.7 yr) completed self-administered TPB questionnaires in 2005 and 2007, respectively.

**Results:**

Both Tanzanian and Sudanese students demonstrated strong intentions to provide care for people with HIV and AIDS. Stepwise linear regression revealed that the TPB accounted for 51% (43% in Tanzania and Sudan) of the variance in intention across study sites. After having controlled for country and past behaviour, the TPB in terms of attitudes, subjective norms and perceived behavioural control accounted for 34% and moral norms for an additional 2,3% of the explainable variance in intention. Across both study sites, attitudes were the strongest predictor of intention followed in descending order by subjective norms, moral norms and perceived behavioural control.

**Conclusion:**

The TPB is applicable to students' care delivery intentions in the context of HIV and AIDS across the two SSA countries investigated. It is suggested that attitudes, subjective norms, moral norms and perceived behavioural control are key factors in students' willingness to treat AIDS and HIV infected patients and should be targets of interventions aimed at improving the quality of health care delivery in this context.

## Background

An estimated 22 million people were living with HIV and AIDS in sub-Saharan Africa, SSA, at the end of 2007 [1]. The United Republic of Tanzania had an estimated 1.6 million people living with HIV and AIDS as of the end of 2003 [1]. Sudan, the biggest country of the SSA continent, is currently recognized by the World Health Organization to suffer an intermediate HIV and AIDS prevalence of 1.6% [1]. The HIV epidemic poses significant development challenges to the low income countries in SSA [2]. It affects the attrition rate among health care workers, their level of motivation, professional practices and absenteeism from work [2]. To date, little is known about how to deal with the deterioration of human resources in the health care systems.

Due to the method of transmission of HIV virus through direct contact with blood, the risk for cross-infection comes into particular focus in medical and dental practices [3]. Over 90% of the HIV infections occurring among health care workers annually stem from developing countries where occupational safety is a neglected issue [4]. Although the risk of transmission in health care settings has been recognized to be low, fear of illness, contagion and death has influenced health workers' attitudes and thus the quality of care provided towards patients with HIV [5-7]. Increased personal risk, lack of necessary skills, knowledge gaps, difficulties in dealing with staff worries and concern about loosing other patients are the most frequent complaints [8-12]. A recent publication focusing on dental students in Khartoum, Sudan, revealed that half of the participants reported a need for further education across HIV and AIDS related issues, suggesting they are not adequately prepared for treating HIV infected patients [8]. Unacceptable knowledge and practice as well as gaps in the availability and access to policies and protocols on the part of health care workers have been observed in several sub-Sahara African countries [11,13-17]. According to the World Health Organization (WHO), dentists have a professional and ethical responsibility to treat patients with HIV and AIDS [18-20]. The importance of training dental and medical staff to provide health care to HIV infected patients at the same level as non-infected people have been widely recognized [17,19]. As future health care workers, the attitudes of dental and medical students towards delivering high quality care for HIV infected patients are of particular concern. Effective promotion of quality health care delivery in the context of HIV and AIDS requires a thorough understanding of the psycho-social determinants of students' intention to provide care to HIV infected patients. Ajzen's theory of planned behaviour (TPB) is a valuable model for identifying the determinants of health behaviours, including quality health care provision for patients with HIV and AIDS [21].

### Theoretical approach

**Figure 1:**
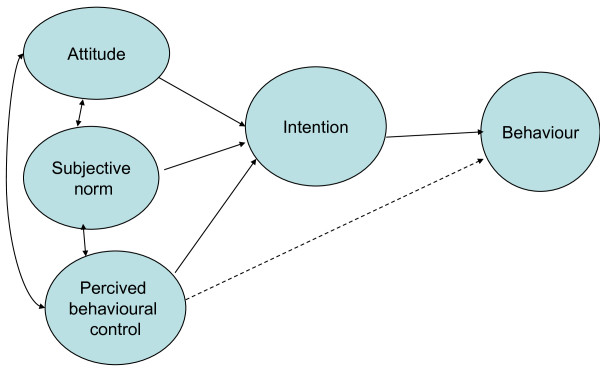
**The theory of planned behaviour (Ajzen 1991)**.

The TPB [21] constitutes a promising framework for understanding and predicting social behaviours (Figure 1). The TPB includes perceived behavioural control on a level with attitude and subjective norm as predictors of behavioural intention. This theory implies that the three predictors influence subsequent behaviour indirectly through behavioural intention. The TPB posits that behavioural intention is a function of attitude, reflecting a favourable or unfavourable evaluation of the particular behaviour and subjective norm, referring to the perceived social pressure to perform the behaviour. Perceived behavioural control reflects the ease or difficulty associated with performance. Attitudes, subjective norms and perceived behavioural control are underpinned by behavioural, normative and control beliefs, respectively. A number of studies have suggested that past behaviour has a residual effect on behavioural intention after the TPB has been taken into account [22,23]. Thus, Ajzen [21] suggested that the TPB is open to the inclusion of additional variables if it can be shown that they capture a significant proportion of the outcome variance.

The TPB has been applied successfully to a range of domains, including HIV related behaviours, particularly condom use [24-28]. With respect to occupational behaviour, the TPB has predicted health workers' use of gloves, their intention to provide home-care for HIV infected patients, their adherence to universal precautions for venipuncture and their intention to provide professional labour support [29-33]. The TPB has been used previously in sub-Saharan African settings to predict HIV protective behaviours, however mostly by small scale studies [27]. It has been advocated that the applicability of socio-cognitive models to the African context should be systematically addressed considering the need for theory-based research in the planning of effective HIV and AIDS related educational programs [34,35].

This study extends analyses of external variables within the TPB in the context of health care delivery by adding past behaviour and moral norms from Triandis' Theory of Interpersonal Behaviour [36]. Personal normative belief or moral norms represents a measure of the personal feelings of moral obligations or responsibility to perform or refuse to perform a given behaviour. Studies of moral norms in the context of the TPB were reviewed by Conner and Armitage [24] who estimated that across investigations moral norms predicted an additional 4% of the variance in intention after controlling for the TPB. Moreover, studies on the Theory of Interpersonal Behaviour have consistently shown that moral considerations are significant predictors of behavioural intentions in the presence of the TPB [37]. Focusing on medical and dental students from Tanzania and Sudan, this study aims to predict the intention to provide surgical treatment to patients with HIV and AIDS as part of future professional work, using the TPB and moral norms. The hypotheses of the present study were:

-attitudes, subjective norms and perceived behavioural control will each contribute positively and statistically significantly to the prediction of intention to provide surgery treatment to HIV and AIDS infected patients

-moral norms will add significantly to the prediction of behavioural intention over and above the TPB

## Methods

### Tanzanian Study group

A cross-sectional survey was carried out from June to September 2005 at Muhimbili University College of Health and Allied Sciences (MUHAS) at the University of Dar-es-Salaam. The target population consisted of students attending the faculties of dentistry and medicine. A total of 1,021 (862 medical and 159 dental) students were enrolled at the college in 2005. Six hundred students (100 students in each study year) attending the 1^st ^to the 5^th ^study year were invited to participate. A total of 476 students agreed to participate and 454 students (response rate 75.6%), mean age 25.6 (sd 2.6), 22.5% females completed supervised self-administered structured questionnaires at the faculty in class-room settings.

### Sudanese Study group

A cross-sectional study was carried out from April to May 2007 among a census of dental students attending dental faculties in Khartoum, the capital city of Sudan. A list of all the dental faculties was obtained from the Ministry of Higher Education and lists of all registered students in their 3^rd^, 4^th ^and 5^th ^years were obtained from all faculties through the Dean's office. The faculties included in this study were both publicly and privately funded. Moreover, they represented all available dental faculties in Sudan, making potentiating admission from all over the country. The total number of dental students registered by the time of the survey was 782 out of which 642 students (response rate 82%), mean age 21.7, (sd 1.9), 82% females completed self-administered, anonymous questionnaires in supervised (by teaching assistants) class-room settings. The main reason for non-participation was absenteeism on the day of the data collection. More information regarding the sampling, recruitment and data collection is available in a previous publication [8].

### Ethical clearance

Written informed consent was obtained from all participants in both countries. A formal ethics waiver was received from the research committee at the University of Science and Technology in Sudan, the Research Committee at MUHAS, Tanzania and from the Regional research committee of Norway (REK VEST).

### Survey instrument

Identical survey instruments were used at both sites. Before administration in the field, the questionnaire was reviewed by experienced local researchers, dental academics and health administrators. The survey instrument was adapted from instruments previously employed in SSA [38]. In Tanzania, the survey instrument was constructed and completed in English. In Sudan, the survey instrument was constructed in English, translated into Arabic (the Sudanese national language) and then back translated into English by independent language experts. The survey instrument covered socio-demographic factors and each component of the TPB developed according to the guidelines proposed by Ajzen and Fishbein [39].

### Variables and measurements

*Intention to provide surgical treatment to patients infected with HIV/AIDS as part of future professional work *was measured by three items. E.g. "How likely is it that you will provide--------". The respondents indicated their subjective probability along a five point response scale from (1) very unlikely to (5) very likely. A sum score was constructed from the three items.

*Attitudes- *were measured by 5 items, three positively worded and two negatively worded. Responses were recorded on a five-point scale ranging from (1) strongly disagree to (5) strongly agree. A sum score was constructed after positively worded items were reversibly scored.

*Subjective norms *were measured by 4 items in relation to all my patients, community leaders, my family and my teacher at the college. E.g. "My teacher at my college wants me to provide surgical treatment to HIV and AIDS infected patients as part of future professional work". Responses were indicated on five-point scales ranging from (1) strongly disagree to (5) strongly agree. A sum score was constructed from the 4 items.

*Perceived behavioural control *was assessed using one item, "How easy or difficult is it for you to provide surgical treatment to patients infected with HIV and AIDS as part of future professional work? Responses were rated on a scale ranging from (1) very difficult to (5) very easy.

*Moral norms *were assessed using 3 items "I I feel guilty if I do not provide ----------". "I get a bad conscience if I do not provide surgical treatment and "It is morally wrong for me not to provide surgical treatment ". Responses were indicated on a scale ranging from (1) strongly disagree to (5) strongly agree. A sum score was constructed from the 3 items.

*Past behaviour *was assessed using a dummy variable in terms of "Have you ever participated in the clinical treatment of HIV and/AIDS infected patients". Answers were provides as (1) yes and (0) no.

### Statistical methods

Confirmatory factor analysis, CFA, with AMOS 16 was employed to test the hypothesized measurement model with respect to intention, attitudes, subjective norms and moral norms, specifying the relationship between the observed variables (indicators) and the underlying latent variables (concepts). Thus CFA was used to test whether the Tanzanian and Sudanese data were consistent with an a priori hypothesized 4-factor model. The parameters of the model were estimated with maximum likelihood (ML) estimation. Missing data were assumed to be missing at random and was handled using the direct approach in AMOS 16 [40].

The adequacy of overall model fit was estimated using chi-square test statistics and the following supplemental fit indices, root-mean-squared error of approximation (RMSEA), the comparative fit index (CFI) and Akaike's information criteria, AIC. In line with the conventional recommendations of Hu and Bentler [41], a good model fit was indicated by a RMSEA less or equal to .06, a CFI greater or equal to .90 and with models having lower AIC being more plausible. The statistical significance of parameter estimates are the critical ratio (CR) representing the parameter estimate divided by its standard error. Based on a level of 0.05, the test statistics need to be <± 1.96 before the null hypothesis can be rejected. All other analyses were performed using SPSS 15.0 (SPSS, Inc, Chicago, Illinois, USA). Internal consistency reliability of-and bivariate associations among the theoretical constructs were assessed using Cronbach's alpha and Pearson's correlation coefficients, respectively. Unadjusted and adjusted marginal means and 95% confidence intervals for the components of the TPB and the construct of moral norm were estimated using General Linear Models, GLM (ANOVA). Linear multiple regression analysis was applied to predict intention from the TPB and external variables. The effect of the independent variables were expressed in terms of standardized regression coefficients (betas) and tested for statistical significance by means of F-test. The fit of the model was reported in terms of the squared multiple correlation coefficient (R^2^).

## Results

### Sample characteristics

Of the 475 Tanzanian students, 17% were in the younger age group (below or equal to 22 yr), 77.5% (368) were males, and 25.4% (121) were in their 5^th ^study year. The corresponding figures pertaining to the Sudanese participants were 69.6% (438), 28% (177) and 36% (231), respectively. Table 1 depicts the percentage distribution of participants according to socio-demographics and country of residence. All socio-demographic characteristics varied substantially and statistically significantly across the two cultures considered.

**Table 1:** Percentage distribution (n) of participating students by socio-demographic characteristics and country.

	Tanzania	Sudan
	**% (n)**	**% (n)**
*Age*		
Younger ≤ 22 yr	17.1 (79)	69.6 (438)
Older >22 yr	82.9 (383)	30.4 (191)**
*Sex*		
Male	77.5 8368)	28.0 (177)
Female	22.5 (107)	72.0 (456)**
*Father's education*		
Low (below university)	52.6 (246)	18.8 (119)
High (university)	47.4 (222)	81.2 (514)**
		
*Mother's education*		
Low (below university)	71.4 (330)	52.9 (334)
High (university)	28.6 (132)	47.1 (297)
*Year of study*		
1^st ^- 4^th ^study year	74.6 (355)	64.0 (410)
5^th ^study years	25.4 (121)	36.0 (231)**
*Preferred future workplace*		
Urban	78.5 (365)	86.7 (540)
Rural	21.5 (100)	13.3 (83)**
Past experience care to HIV infected patients		
No	59.5 (270)	90.9 (501)
Yes	40.5 (184)	9.1 (50)**

**p < 0.001 *p < 0.05

### Measurement model for the extended TPB

CFA indicated that an initially proposed 4-factor model specified for the extended TPB questionnaire was not an acceptable fit on any indices employed, neither with the Tanzanian (χ^2 ^= 308,057, df = 84, CFI = 0.91, RMSEA = 0.07, AIC = 380.0) nor with the Sudanese data (χ^2 ^= 351,398, df= 84, CFI = 0.89, RMSEA = 0.07, AIC = 453.3). Modification indices indicated some specified error terms to co-vary and two cross loading from subjective norms and moral norms to indicators of intention. Re-running the model with the above suggested modifications indicated acceptable fit for the Tanzanian (χ^2 ^= 191,735 df = 79, CFI = 0.96, RMSEA = 0.05, AIC = 273.7) as wells to the Sudanese data (χ^2 ^= 281.058, df = 83, CFI = 0.92, RMSEA = 0.06, AIC = 385.0). All factor loadings (standardized regression weights) were in the expected direction and were statistically significant at CR>1.96, with inter-factor correlations ranging from 0.46-0.78 (Tanzania) and from 0.50-0.68 (Sudan). Thus, all inter-factor correlations were below the threshold of 0.80 which is set as cut off to indicate poor discriminative validity [41].

### Descriptive statistics

Table 2 depicts unadjusted and adjusted marginal means and 95% confidence intervals for the components of the TPB and the construct of moral norm by country of residence. Country differences were estimated after controlling for potential confounding effect from socio-demographic variables (age, gender, parental education, study year) using General Linear Models, GLM (ANOVA). In both countries, students had on average positive attitudes, strong moral norms and strong intentions regarding care delivery to patients with HIV and AIDS. Both groups of students had on average moderately strong subjective norms and perceived less control regarding this behaviour. Tanzanian students had on average more positive attitudes and stronger intentions, perceived control, moral norms and subjective norms compared to their Sudanese counterparts. Internal consistency reliability in terms of Cronbach's alpha ranged from 0.83 (moral norms/subjective norms) to 0.44 (attitudes) in Tanzania and from 0.81 (attitudes/intention) to 0.45 (subjective norms) in Sudan.

**Table 2:** Unadjusted (sd) and adjusted estimated marginal means (95% CI) and Cronbach's alpha for the TPB constructs and moral norms according to country.

	Tanzania			Sudan		
**Tanzania**	**Mean (sd)** **[Range] ^a^**	**Mean (95% CI)** **[Range] ^b^**	**Cronbach's alpha**	**Mean (sd)** **[Range] ^a^**	**Mean (95% CI)** **[range] ^b^**	**Cronbach's alpha**

Attitudes (5 item)	18.9 (2.8) [9-25]	19.2 (18.8-19.5)** [9-25]	0.44	16.1 (2.7) [5-24]	16.0 (15.6-16.3**) [5-25]	0.81
Subjective norms (5 items)	14.5 (3.2) [4-20]	14.6 (14.0-14.8)** [4-20]	0.83	12.5 (2.7) [4-19]	12.5 (12.2-12.9) [4-19]	0.45
Moral norms (3 items)	11.7 (2.6) [3-15]	11.8 (11.5-12.1)** [3-15]	0.83	10.6 (2.8) [3-15]	11.0 (10.6-11.3) [3-15]	0.60
Perceived control (1 item)	3.2 (1.1) [1-5]	3.2 (3.1-3.3)** [1-5]	-	2.4 (1.0) [1-5]	2.6 (2.4-2.6) [1-5]	
Intention (3 items)	12.2 (2.2) [3-15]	12.3 (12.0-12.6)** [3-15]	0.82	10.0 (2.9) [3-15]	10.3 (9.9-10.6) [3-15]	0.81

^a ^Unadjusted means

^b ^Adjusted for age, gender, parental education and study year

### Prediction of intention to provide surgical treatment to patients with HIV and AIDS as part of future professional work

In Tanzanian and Sudanese students, the TPB components, moral norms, and past behaviour were statistically significantly associated with intention. In Tanzanian students, Pearson's correlation coefficients ranged from r = .52 between attitudes and intention to r = .14 between past behaviour and intention. In Sudanese students, Pearson's correlation ranged from r = .54 between intention and attitudes to r = .03 between intention and past behaviour.

Table 3 depicts the results from hierarchical linear regression analysis assessing the fit of the extended TPB model among Tanzanian and Sudanese students. Country and past behaviour were entered in the first step explaining 15.7% (R^2 ^change = 0.157, p < 0.001) of the variance in intention. Adding attitudes, subjective norms and perceived behavioural control in step 2 increased the explained variance by 33.4% (R^2^change = 0.334 p < 0.001). Moral norm added in step 3 raised the explained variance by 2.3% (R^2 ^change = 0.023 p < 0.001). A total of 6 variables accounted for 51.4% of the variance in intention (43.7% and 43.9% in Tanzania and Sudan, respectively). In the final equation, attitude was by far the strongest predictor of intention (beta = 0.35), followed in descending order by subjective norms (beta= 0.22), moral norm (beta= 0.17), perceived behavioural control (beta = 0.16) and past experience (beta = 0.06). The effect of country (beta = 0.35, p < 0.001) in step 1 was reduced to (beta = 0.04, p = 0.099) in the final third step, whereas the strength of the effect from past behaviour was maintained from step 1 (beta= 0.08, p < 0.001) to step 3 (beta= 0.06, p < 0.001). Statistically significant two-way interactions occurred, in terms of country × attitudes (R^2 ^change 0.007, F change = 13.7, p < 0.001) and country × subjective norms (R^2 ^change 0.009, F change = 17.0, p < 0.001). Stratified analyses suggested that the relationship between attitude and intention and between subjective norms and intention were statistically significantly stronger in Sudanese- than in Tanzanian students.

**Table 3:** Intention to provide surgical treatment to HIV and AIDS patients by attitudes, subjective norms, perceived behavioural control and moral norms.

Model	R	R^2^	R^2 ^change	Beta^a^
*Step 1*				
Country				-0.05ns
Past behaviour	0.39	0.157**	0.157**	0.06*
*Step2*				
Attitudes				0.35**
Subjective norms				0.22**
Perceived behavioural control	0.70	0.491**	0.334**	0.16**
*Step 3*				
Moral norm	0.71	0.51**	0.023**	0.17**

** p < 0.001, * p < 0.05

^a ^Standardized regression coefficients (beta), final step

## Discussion

This study supports the applicability of an extended version of the TPB to students' intended care delivery for patients with HIV and AIDS in two culturally different sub-Saharan African countries. According to the CFA, the extended TPB questionnaire reflected four concepts across the study sites in terms of attitudes, subjective norms, moral norms and intention, thus lending support to it's within construct validity, formally. This appears to imply that the four constructs underlying the TPB questionnaire are discrete measures that can be reliably assessed in Tanzanian and Sudanese students and that those measures can be reported as four summary scores.

A total of six variables explained 51% of the variance in health care delivery intentions across study sites. After having controlled for country and past behaviour, attitudes, subjective norms and perceived behavioural control accounted for an additional 34% of the explainable variance in intention. This finding is consistent with those of previous studies, whereby the TPB has explained 68% of nurses' intention to adhere to universal precautions, 48% of health workers' intention to provide home care for HIV infected individuals, 35% of nurses' intention to care for SARS patients and 70% of nurses intended labour support [29-33]. In line with a growing body of research supporting the role of perceived moral obligations as an independent predictor of intention, moral norms contributed 2.3% to the explained variance in students' intentions after controlling for the TPB variables. This factor which has moral connotations and represents personal feelings of responsibility has been considered to be important in the adoption of several health related behaviours [36,37]. Conner and Armitage [25] found that moral norms contributed an additional 4% of the variance in intention after controlling for the TPB across various behaviours. The present results suggest that the TPB and its extended version is useful in the SSA context in terms of identifying correlates of health care delivery that can be targets in interventions aimed at improving health care delivery to HIV infected patients.

A major shortcoming of the TPB model has been its inability to account for the influence of past behaviour [42,43]. Evidence from meta-analytical reviews suggests that the addition of past behaviour to the TPB explains on average 7% of the variance in intention [25]. In this study, past experience significantly predicted intention after controlling for the TPB and moral norms and left the cognitive variables of the model almost unaffected. This suggests that the TPB provides a fairly accurate description of the intention formation process considering HIV and AIDS related care delivery among Tanzanian and Sudanese students. Students decide upon care delivery for HIV infected patients mainly as a consequence of situation specific expectations of the behaviour itself and to a lesser extent because they have engaged in similar care delivery previously.

Tanzanian and Sudanese students had on average strong intentions to provide surgical treatment to HIV infected patients as part of their future professional work. In contrast, in a study of Taiwanese nurse students' care intentions, almost all stated that they did not intend to care for HIV infected patients [44]. In this study, intended health care delivery was primarily driven by attitudes followed in descending order of importance by social norms, moral norms and perceived behavioural control in both countries. This finding is congruent with findings in other studies of treatment delivery and compliance with precautions in health care workers [29-32]. This finding is also similar to that reported by Sauls [33], who identified attitudes as more influential in determining health care delivery intentions than subjective norms. In contrast, Vermette and Godin [30] found perceived behavioural control to be the strongest influencing factor of nurses' intention to provide home care for HIV infected people. Students who did not express confidence in their ability to circumvent difficulties associated with care delivery, who evaluated care delivery negatively and who felt less normative pressure from colleagues at the faculty and less moral obligations to act were less likely to have strong intentions. These findings add insight to faculty administrators and educators to further develop strategies to increase students' intention to care for patients with HIV and AIDS. Providing sufficient and adequate protective equipments, routinely practicing infection control measures and protocols and providing up to date continuing education and training exercises should improve students' ability to overcome perceived obstacles related to care delivery. Even more important is the enhancement and reinforcement of students' positive attitudes through verbal expression of approval, persuasive messages based on strong arguments, substantial rewards and psychological support by faculty staff to encourage and acknowledge their efforts. Students' decision to provide surgical treatment to HIV infected patients was also influenced by their expectations that faculty colleagues would approve their provision of such treatment. In planning intervention programs, one approach which could be particularly important among students having less care delivery experience is to train peer leaders to communicate the importance of quality care for HIV infected patients. Interventions might also benefit from making students' focus on moral obligations by increasing their awareness of others' needs and their perception that providing quality care could help relieve such needs.

Some limitations of this study should be considered when drawing inferences based upon its results. In Tanzania, dental and medical students were recruited from one university, thus the representativeness of the findings to other undergraduate- and post-graduate students is unknown. Self-selection might also be a potential limitation since the students who chose to participate might differ from those who did not implying that only students with interest in health care delivery for HIV infected patients responded to the survey invitation. Students might have over reported intention to provide surgical treatment to HIV infected patients because of social desirability bias. However, a general effect of low reliability is weak associations between variables. Thus, the magnitude of the correlations presented, and the findings harmonizing with the TPB indicate acceptable reliability as well as validity of the results. Past experience as assessed in this study was limited to the extent that it provided no information about the level and frequency of HIV and AIDS related care delivery. Whereas the present findings support the notion that the TPB model is applicable in the sub-Saharan African context, it says nothing about the validity of the model per se, only that the TPB is just as useful in sub Saharan Africa as in other industrialized country contexts.

## Conclusion

The TPB is applicable to students' intention to provide health care to patients with HIV and AIDS across two sub-Saharan African countries. It is suggested that attitudes, subjective norms, moral norms and perceived behavioural control are key factors in students' decision to treat HIV infected patients and should be targeted in interventions aimed at improving health care quality in the context of HIV and AIDS.

## Competing interests

The authors declare that they have no competing interests.

## Authors' contributions

ANÅ: Principle investigator at both sites who conceived of the study, analyzed the data and did the manuscript writing. EN: Participated in the design and analyses of the Sudanese data. Responsible for collecting the Sudanese data.

## Pre-publication history

The pre-publication history for this paper can be accessed here:

http://www.biomedcentral.com/1472-6963/9/213/prepub
